# Supplementary material for: Exosomal Profiling Reveals Mechanisms of Hibernation-Associated Neuroprotection
Source: bioRxiv. 2026 Jun 29:2026.06.23.733742. Preprint. [Version 1] doi: 10.64898/2026.06.23.733742 (PMC13345078; doi:10.64898/2026.06.23.733742)
Supplement: Supplement 9 [file NIHPP2026.06.23.733742v1-supplement-9.pdf]

bioRxiv preprint doi: https://doi.org/10.64898/2026.06.23.733742; this version posted June 29, 2026. The copyright holder for this preprint (which was not certified by peer review) is the author/funder. This article is a US Government work. It is not subject to copyright under 17 USC 105 and is also made available for use under a CC0 license.

Supplementary Material

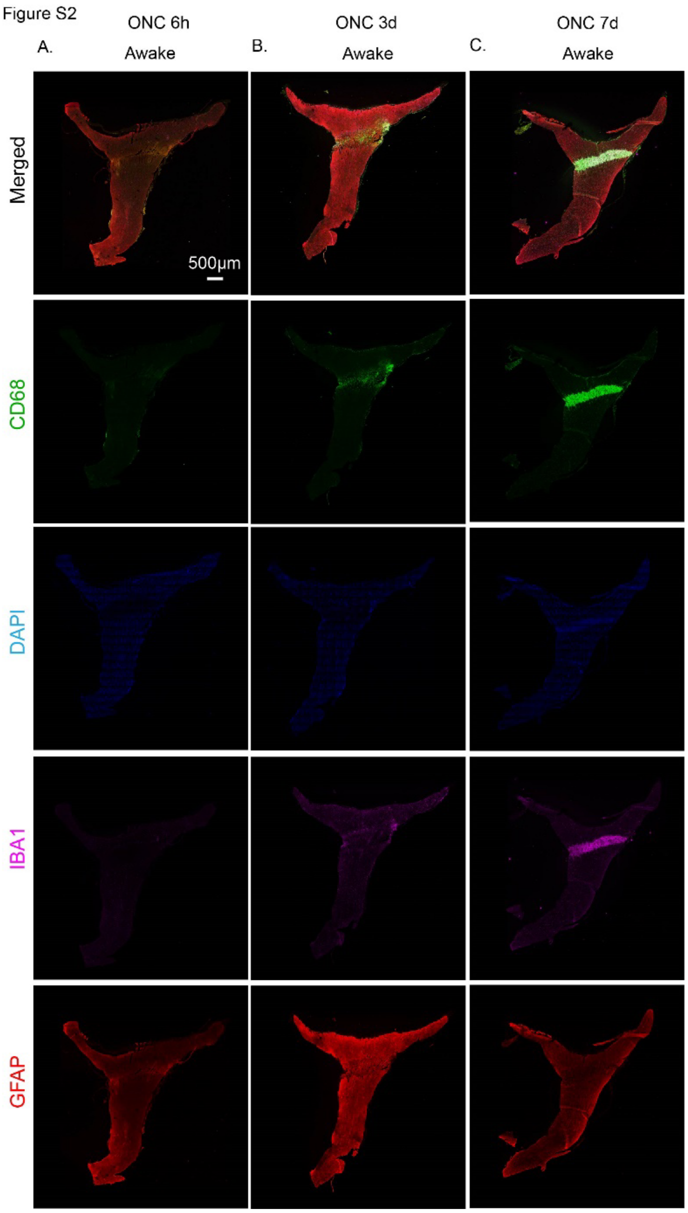

Supplementary Figure S1. Confocal images of representative optic nerves from Awake TLGS following optic nerve crush, collected at 6 h (A), 3 d (B), 7 d (C) post-injury. Optic nerves were stained for CD68 (green), IBA1 (magenta), GFAP (red), and counterstained with DAPI (blue). Scale bar: 500 µm.

bioRxiv preprint doi: https://doi.org/10.64898/2026.06.23.733742; this version posted June 29, 2026. The copyright holder for this preprint (which was not certified by peer review) is the author/funder. This article is a US Government work. It is not subject to copyright under 17 USC 105 and is also made available for use under a CC0 license.

Figure S2

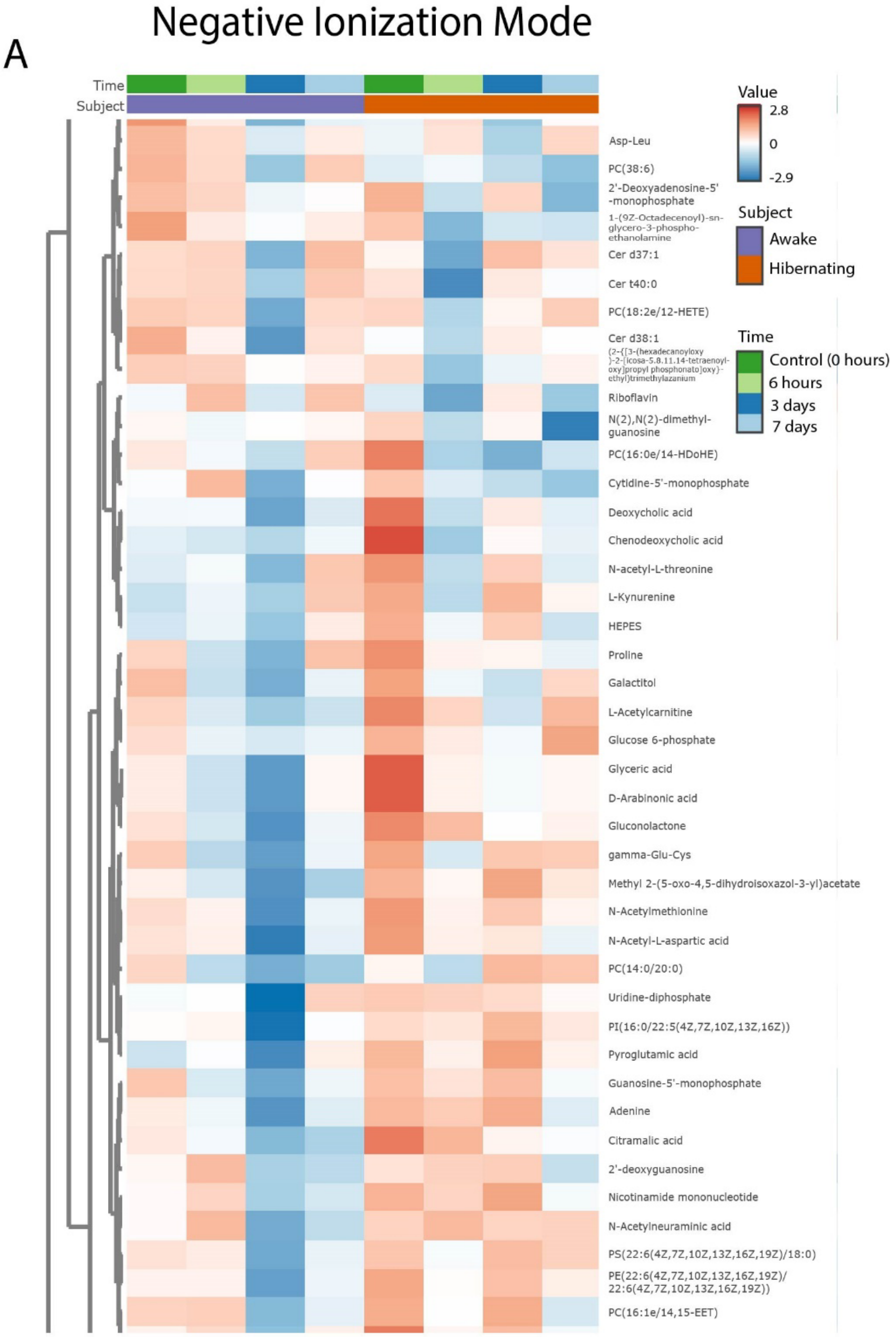

Supplementary Figure S2. Heatmap of additional metabolites identified in negative ionization mode. Metabolites in this group exhibit variable changes following injury.

bioRxiv preprint doi: https://doi.org/10.64898/2026.06.23.733742; this version posted June 29, 2026. The copyright holder for this preprint (which was not certified by peer review) is the author/funder. This article is a US Government work. It is not subject to copyright under 17 USC 105 and is also made available for use under a CC0 license.

Figure S3

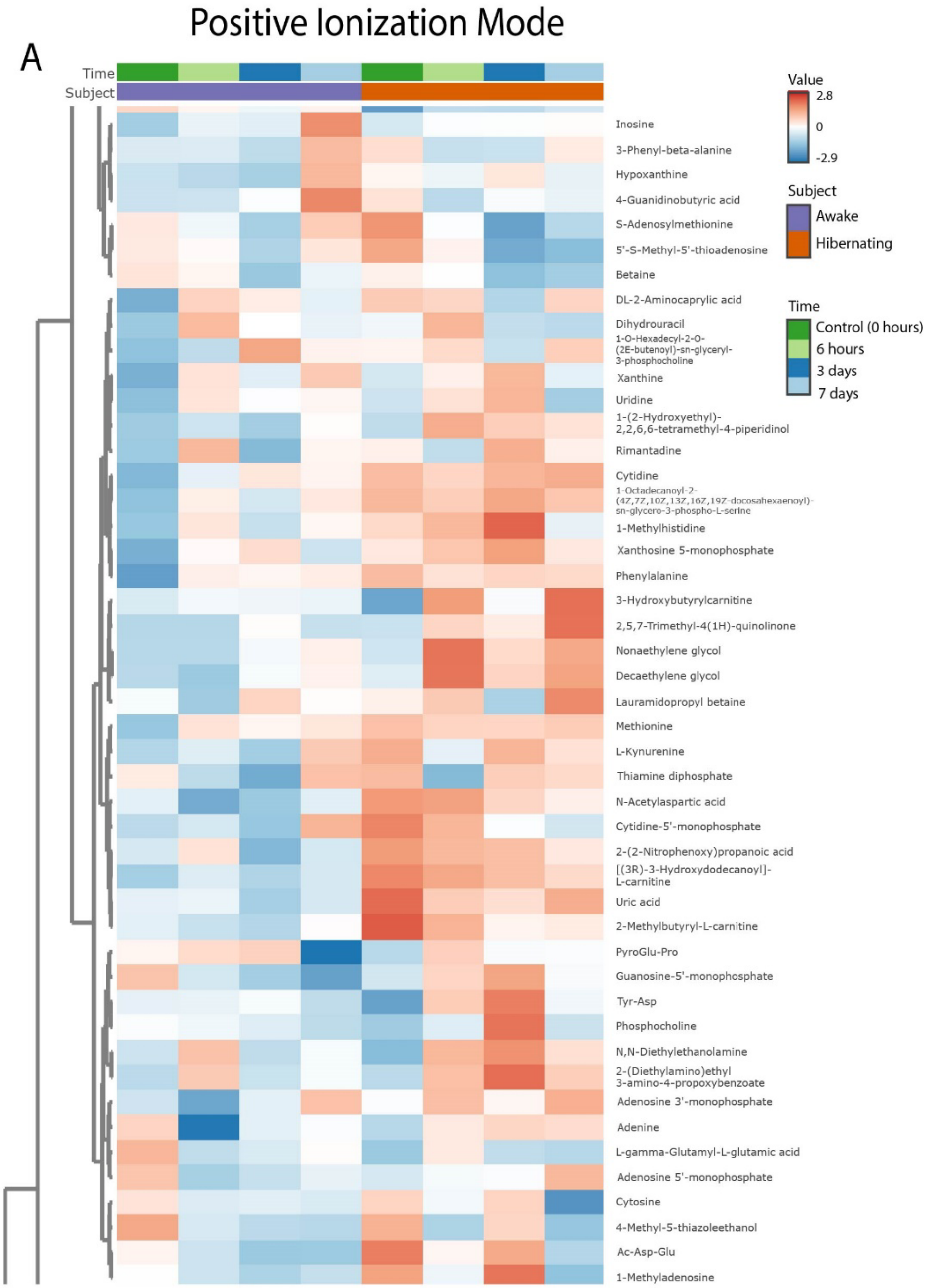

Supplementary Figure S3. Heatmap of additional metabolites identified in positive ionization mode. Metabolites in this group exhibit variable changes following injury

bioRxiv preprint doi: https://doi.org/10.64898/2026.06.23.733742; this version posted June 29, 2026. The copyright holder for this preprint (which was not certified by peer review) is the author/funder. This article is a US Government work. It is not subject to copyright under 17 USC 105 and is also made available for use under a CC0 license.

Supplementary Methods

Optic Nerve Crush

Squirrels were anesthetized using isoflurane gas, with induction at 5% and maintenance at 4% via a low-profile nose cone. Anesthetic gas was scavenged using an attached activated charcoal filter. Sterile surgical instruments were autoclaved prior to use and subsequently disinfected between animals by immersion in 70% ethanol followed by bead sterilization at approximately 270°C for 20 seconds. To prevent infection, topical antibiotic (polymyxin B-neomycin-bacitracin) ophthalmic ointment was applied to the surgical eye. To prevent corneal dehydration during anesthesia, Systane® lubricating gel was applied to both eyes. Ketoprofen (5 mg/kg, subcutaneous) was administered prior to recovery to provide preemptive analgesia, which helps reduce the severity and duration of postoperative pain.

For hibernating animals, body temperature was maintained at approximately 4°C using an ice pack placed beneath the animal during the procedure. Animals were anesthetized with isoflurane (induction at 3%, and maintenance at 3%) to sustain the hibernation state and prevent arousal. Animals were kept under isoflurane anesthesia to maintain the hibernation state and prevent arousal. To minimize sensory stimulation, ambient lights were turned off, the room was isolated from external noise, and exposure to illumination from the surgical microscope was kept to a minimum. To maintain body temperature during transfer and to minimize light, animals were transferred to and from the hibernaculum in a foam shipping container equipped with ice packs. In the fall preceding the hibernation season, animals were implanted subcutaneously with temperature transmitters (TA-F40, Data Sciences International) or iButtons (AlphaMach), enabling wireless body temperature monitoring to confirm that animals remained in torpor following the surgical procedures [73].

bioRxiv preprint doi: https://doi.org/10.64898/2026.06.23.733742; this version posted June 29, 2026. The copyright holder for this preprint (which was not certified by peer review) is the author/funder. This article is a US Government work. It is not subject to copyright under 17 USC 105 and is also made available for use under a CC0 license.

For awake, normothermic animals, a disposable hand warmer wrapped in a paper towel was used to provide gentle warmth while preventing direct skin contact.

Under a dissection stereo microscope, a temporal canthotomy was performed on one eye, followed by a lateral conjunctival incision near the corneal margin. The retinal blood supply was preserved throughout the procedure. After gently separating the retractor bulbi muscles, the optic nerve was exposed intraorbitally using blunt dissection. A calibrated cross-action forceps was used to compress the optic nerve for 10 seconds, approximately 1–2 mm posterior to the globe.

After the injury was induced, the surgical site was closed with sutures. Animals were monitored continuously throughout recovery from anesthesia and returned to their home cages once fully ambulatory. Postoperative monitoring included assessment for signs of pain or distress. Any animal exhibiting signs of discomfort was evaluated in consultation with veterinary staff and treated or euthanized as necessary.

Experimental endpoints for the metabolomics study were at 6 hours, 3 days, or 7 days post-injury. For anatomical assessments, additional survival time points included 14 days post-injury.

Immunohistochemistry: Retina

Eyes were fixed in 4% PFA for 1 hour followed by dissection in PBS. Using a binocular dissecting scope, the anterior part of the eye is removed by first making an incision with a razor blade and then inserting dissection scissors to remove the lens and cornea. The eyecup is then cut into a 4-petal flower shape by making radial incisions at 90-degree intervals. Using fine forceps, the retina was carefully lifted away from the underlying retinal pigment epithelium. Special care must be taken to gently cut the retina free from the optic nerve head as the optic nerve in squirrels is oriented horizontally.

bioRxiv preprint doi: https://doi.org/10.64898/2026.06.23.733742; this version posted June 29, 2026. The copyright holder for this preprint (which was not certified by peer review) is the author/funder. This article is a US Government work. It is not subject to copyright under 17 USC 105 and is also made available for use under a CC0 license.

After carefully removing the vitreous using fine forceps and a soft brush, retinas were permeabilized and blocked in 2 % normal goat serum (NGS) with Triton X-100 in PBS for 30 min, replacing the solution every 10 min [74]. Tissues were then incubated at 4 °C for 7 days with a primary antibody against antibrain-specific homeobox/POU domain protein 3A (Brn3a, red, 1:750, Cat#: C-20 [discontinued]; Santa Cruz Biotechnologies) or RNA Binding Protein, Multi-Splicing (RBPMS, red, 1:500, Cat#: GTX118619; Genetex). Following thorough PBS washes, retinas were incubated with the appropriate Cy3-conjugated donkey secondary antibody overnight at room temperature.

Immunohistochemistry: Optic nerve

Optic nerves were fixed in 4% PFA for 1 hour at room temperature, followed by three washes in PBS. Fixed tissues were embedded in OCT compound and 30-um thick cryosections were collected using a cryostat (Leica, Nussloch, Germany). Cryosections were incubated with antibodies for CD68 (green, Biorad, Cat#: 019-19741, 1:100), IBA1 (magenta, Wako, Cat#: 019-19741, 1:100), and glial fibrillary acidic protein (GFAP, red, Aves Labs, Cat#: GFAP87987979, 1:500). Nuclei were counterstained with DAPI (blue, 1:2000). After staining, sections were mounted on glass slides using antifade mounting medium and imaged using a Zeiss LSM 780 confocal microscope.

Gene expression analysis

RT-qPCR was used to quantify gene expression changes in BV2 microglial cells. BV2 microglial cells were treated with various reagents (e.g., LPS or treated with TLGS-derived exosomes) for 6 hours, after which cells were collected for RNA extraction and subsequent RT-PCR analysis to quantify gene expression changes. Total RNA was extracted using NuceloSpin RNA, Mini kit for RNA purification (Takara Bio, Cat: 740955.50) and reverse-transcribed to

bioRxiv preprint doi: https://doi.org/10.64898/2026.06.23.733742; this version posted June 29, 2026. The copyright holder for this preprint (which was not certified by peer review) is the author/funder. This article is a US Government work. It is not subject to copyright under 17 USC 105 and is also made available for use under a CC0 license.

cDNA using (Takara PrimeScript 1st strand cDNA synthesis kit (Takara Bio, Cat: 6110A) according to manufacturer's instructions. cDNA was used for RT-qPCR in a reaction volume of 15µl containing, 2µL cDNA, 2µl of primer mixture, and 7.5 µl of Master mix (EmeraldAmp® GT PCR Master Mix, Takara Bio, Cat: RR310B) and 3.5 µl of water. RT-qPCR was performed using a CFX96 Real-Time PCR Detection System (Bio-Rad). Ribosomal protein S14 (RPS14) was used as an internal control. mRNA expression levels were normalized to control samples using the comparative Ct ($2^{-\Delta\Delta Ct}$) method. A list of primers is shown below. At least 3 replicates of each experiment were performed.

Primers:

TNF-α-662	CCAGGAGAAAGTCAACCTCC
TNF-α-870	GAGCAATGACTCCAAAGTAGAC
iNOS-1531	CTCCACGGGCCCGGTACTCA
iNOS-1337	TCCTGGAGGAAGTGGGCCGAA
RPS14-548	CACAGACGGCGACCACGAC
RPS14-363	CACTGCCCTGCACATCAAACT

Statistical analysis

Statistical analyses were performed using GraphPad Prism, Microsoft Excel, or custom MATLAB scripts. Pairwise comparisons were conducted using paired t-tests, while comparisons among three or more groups were performed using one-way analysis of variance (ANOVA).
